# *Streptococcus mutans* Membrane Vesicles Harboring Glucosyltransferases Augment *Candida albicans* Biofilm Development

**DOI:** 10.3389/fmicb.2020.581184

**Published:** 2020-09-11

**Authors:** Ruixue Wu, Ye Tao, Yina Cao, Yan Zhou, Huancai Lin

**Affiliations:** ^1^Department of Preventive Dentistry, Hospital of Stomatology, Guanghua School of Stomatology, Sun Yat-sen University, Guangzhou, China; ^2^Guangdong Provincial Key Laboratory of Stomatology, Sun Yat-sen University, Guangzhou, China

**Keywords:** *Streptococcus mutans*, *Candida albicans*, membrane vesicles, glucosyltransferases, exopolysaccharides

## Abstract

*Candida albicans*, as the most common fungus in the oral cavity, is often detected in early childhood caries. *Streptococcus mutans* is the major etiological agent of dental caries, but the role of *S. mutans* on *C. albicans* growth and biofilm development remains to be elucidated. Membrane vesicles (MVs) are a cell-secreted subcellular fraction that play an important role in intercellular communication and disease progression. In the present study, we investigated whether MVs from *S. mutans* augment *C. albicans* growth and biofilm development. The results indicated that *S. mutans* MVs augmented *C. albicans* biofilm development but had no significant effect on *C. albicans* growth under planktonic conditions. Subsequently, we labeled *S. mutans* MVs with PKH26 and used confocal laser scanning microscopy (CLSM) to track *S. mutans* MVs, which were observed to be located in the *C. albicans* biofilm extracellular matrix. Monosaccharide tests showed that *S. mutans* MVs contribute to sucrose metabolism in *C. albicans*. Polysaccharides were significantly enriched in the *S. mutans* MV-treated group. MVs from Δ*gtfBC* mutant strains were compared with those from the wild-type *S. mutans.* The results revealed that MVs from the Δ*gtfBC* mutant had no effect on *C. albicans* biofilm formation and exopolysaccharide production. In addition, *C. albicans* biofilm transcriptional regulators (*Ndt80*, *Als1*, *Mnn9*, *Van1*, *Pmr1*, *Gca1*, and *Big1*) expression were upregulated in *S. mutans* MV-treated group. In summary, the results of the present study showed that *S. mutans* MVs harboring glucosyltransferases involved in exopolysaccharide production augment *C. albicans* biofilm development, revealing a key role for *S. mutans* MVs in cross-kingdom interactions between *S. mutans* and *C. albicans*.

## Introduction

Early childhood caries (ECC) exhibits high infectivity and is one of the most common diseases in children worldwide (Pitts et al., 2017). According to the results of the 4th National Oral Health Survey of China in 2015, the prevalence of dental caries in 5-year-old children was 71.9%, whereas a prevalence of 66.1% was observed in 2005 (Du et al., 2018). *Streptococcus mutans* is a Gram-positive bacterium that has a strong ability to produce acid and is considered a major etiologic agent of ECC (Hajishengallis et al., 2017). However, other oral microorganisms also contribute to ECC progression (Hajishengallis et al., 2017). *Candida albicans*, an opportunistic pathogen, is the most common fungus in the oral cavity (Simon-Soro et al., 2013). A number of studies have reported that *C. albicans* can often be detected with *S. mutans* in ECC dental plaque samples, and the detection rate of *C. albicans* is significantly higher in children with ECC than in caries-free children (Yang et al., 2012; Xiao et al., 2018a, b). High-throughput amplicon sequencing was used to characterize the oral microbiome associated with ECC, and the results indicated that *C. albicans* was the most abundant fungus and correlated with caries (O’Connell et al., 2020). Therefore, *C. albicans*, as a part of dental plaque, can enhance the virulence of plaque biofilms and is considered to be an important factor in ECC (Hajishengallis et al., 2017; Koo et al., 2018).

In recent years, the cross-kingdom interaction between *S. mutans* and *C. albicans* has drawn a great deal of attention, with *S. mutans* having been shown to play an important role in the biofilm formation of *C. albicans*. For instance, *S. mutans*-derived glucosyltransferase B (GtfB) can be absorbed to the *C. albicans* cell surface and bind to *C. albicans* surface mannan to promote *C. albicans* biofilm formation (Gregoire et al., 2011; Hwang et al., 2015; Ellepola et al., 2017). Moreover, *S. mutans* exopolysaccharide can enhance the antifungal drug tolerance of *C. albicans* biofilms (Kim et al., 2018). Antigen I/II on the *S. mutans* surface can mediate adhesion between *S. mutans* and *C. albicans* and increase acid production within the biofilm (Yang et al., 2018). However, it has also been reported that mucatin, competence-stimulating peptide and subproducts of *S. mutans* suppress *C. albicans* hyphal cell and biofilm formation (Jarosz et al., 2009; Joyner et al., 2010; Barbosa et al., 2016). Thus, there is a tightly regulated cooperative-antagonistic balance between *S. mutans* and *C. albicans*, and when this balance is disrupted, the synergy between the two microorganisms will contribute to the development of ECC (Koo et al., 2018). The role of *S. mutans* in the growth and biofilm development of *C. albicans* is important and complex, and the mechanism of this cross-kingdom interaction remains unclear.

Membrane vesicles (MVs) are a cell-secreted subcellular fraction with sizes ranging from 20 to 500 nm (Toyofuku et al., 2019). Due to the misconception that the thick cell wall of Gram-positive bacteria prevents the formation of MVs, Gram-positive bacterial MVs did not draw attention until 1990 (Dorward and Garon, 1990). In recent years, MV production has been reported in Gram-positive bacteria, such as *Streptomyces lividans*, *Staphylococcus aureus*, and *Bacillus subtilis* (Brown et al., 2014; Schrempf and Merling, 2015; Im et al., 2017). Gram-positive bacterial MVs consist of lipid bilayers and play a role in microbial adhesion, material exchange and competition (Brown et al., 2015; Liu et al., 2018). In 2014, MVs were successfully isolated from *S. mutans* culture supernatant and characterized, setting the stage for the study of *S. mutans* MVs (Liao et al., 2014). *S. mutans* MVs contain biologically active substances, such as extracellular DNA (eDNA) and proteins (Liao et al., 2014; Rainey et al., 2019; Senpuku et al., 2019), and *S. mutans* MVs have been demonstrated to deliver eDNA, which contributing to *S. mutans* biofilm development (Liao et al., 2014; Rainey et al., 2019). Moreover, *S. mutans* MVs can enhance the biofilm formation of other oral microorganisms, including *Streptococcus sanguinis*, *Streptococcus mitis*, *Streptococcus gordonii*, and *Streptococcus oralis* (Senpuku et al., 2019). However, the mechanism associated with the phenomenon is still unknown.

In the present study, we investigated the role of *S. mutans* MVs on *C. albicans* growth and biofilm development. We used ultracentrifugation to isolate *S. mutans* MVs and evaluated their effect on *C. albicans* growth and biofilm formation. In addition, we analyzed the mechanism by which *S. mutans* MVs affect *C. albicans* biofilm development. Our findings will provide new insights into the cross-kingdom interaction between *S. mutans* and *C. albicans*, which may be a target for ECC prevention.

## Results

### Morphological Characterization of *S. mutans* MVs

**FIGURE 1:**
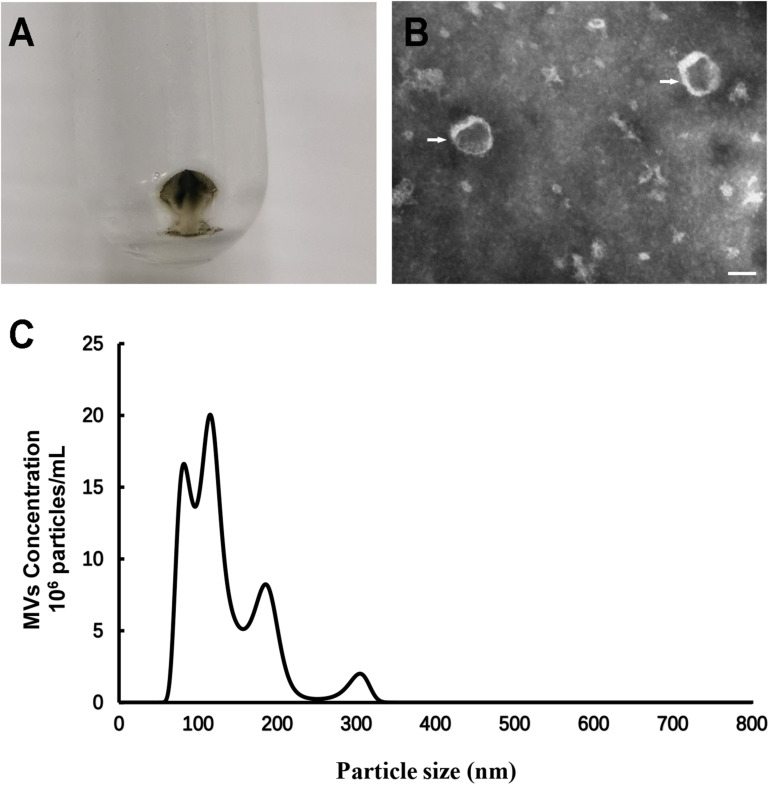
Morphological and dimensional aspects of *S. mutans* MVs. **(A)** *S. mutans* MVs were isolated by ultracentrifugation. **(B)** Negative staining TEM of *S. mutans* MVs. The white arrows indicate the *S. mutans* MVs, Scale bar, 50 nm. **(C)** Size distribution of *S. mutans* MVs evaluated by NTA.

Centrifugation of *S. mutans* culture medium produced black-brown vesicle pellets (Figure 1A). Negative staining transmission electron microscopy (TEM) analysis of *S. mutans* MVs showed the presence of spherical structures in which the individual membrane units could not be observed (Figure 1B). Nanoparticle tracking analysis (NTA) was performed to measure the size of *S. mutans* MVs, and the results demonstrated that the diameter of *S. mutans* MVs was 151.30 ± 15.16 nm (Figure 1C). The yield of *S. mutans* MVs was 1.31 ± 0.23 pg/colony-forming unit (CFU).

### *S. mutans* MVs Augment *C. albicans* Biofilm Formation

**FIGURE 2:**
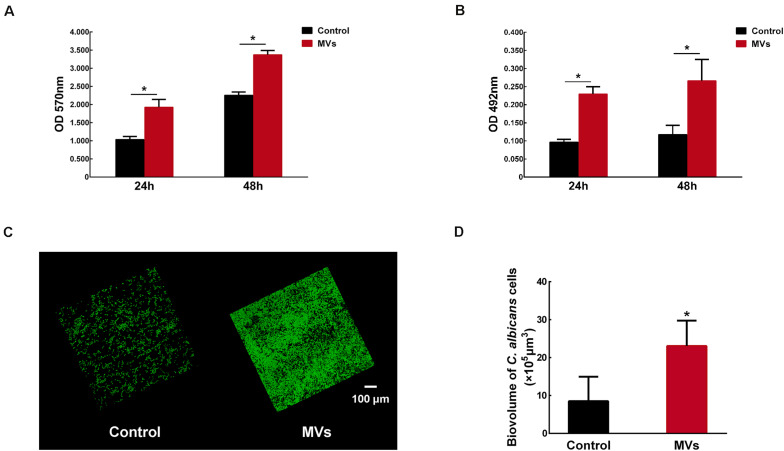
The effect of *S. mutans* MVs on *C. albicans* biofilm formation. **(A)** Crystal violet assay of *C. albicans* biofilms. **(B)** XTT assay of *C. albicans* biofilms. **(C)** Representative CLSM images of *C. albicans* 24-h-old biofilms. Images were taken at 20 × magnification, and the *C. albicans* cells were stained with SYTO-9 (in green). **(D)** The biovolume of *C. albicans* from CLSM images. The experiments were performed in three distinct replicates, and the data are presented as the means ± SD, *P < 0.05 vs control group, using PBS as control group.

**FIGURE 3:**
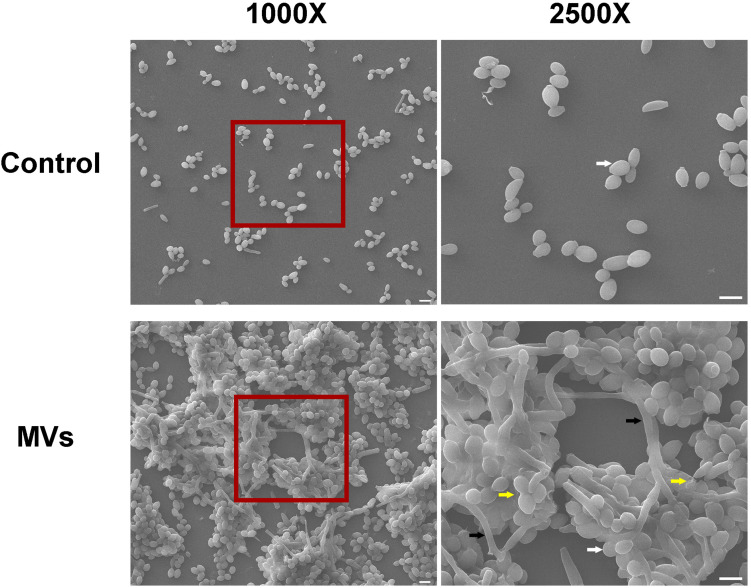
Morphological characteristics of *C. albicans* 24-h-old biofilms. Each field of vision was magnified 1,000 × and 2,500 ×. The red boxes indicate the magnified viewing area. The white arrows show the yeast cells, the black arrows show the hyphal cells, and the yellow arrows indicates the biofilm extracellular matrix. Scale bar, 5 μm.

*Candida albicans* 24-h biofilm formation was assessed in the presence of 40 μg/mL *S. mutans* MVs, a concentration that was selected following a dose-dependent study (Supplementary Figure 1). A crystal violet assay was used to quantify *C. albicans* biofilm biomass after incubation with *S. mutans* MVs. The results showed that the biofilm biomass of *C. albicans* at 24 and 48 h was significantly increased compared to that of the control group (Figure 2A; *P* < 0.001), particularly in the early stage (24 h) of biofilm maturation, with an increase of approximately 1.86-fold observed compared to that of the control group. Similar to the crystal violet assay results, an XTT reduction assay revealed the same pattern for *C. albicans* biofilm viability (Figure 2B; *P* < 0.01). Confocal laser scanning microscopy (CLSM) showed that *S. mutans* MVs enhanced the clustering and accumulation of *C. albicans* under biofilm-forming conditions (Figure 2C). The biovolume of *C. albicans* cells was calculated using COMSTAT software. The *C. albicans* cell biovolume in the *S. mutans* MV-treated group was significantly increased by 1.70-fold compared to that observed in the control group (Figure 2D; *P* < 0.001), which was corroborated by subsequent findings. Furthermore, scanning electron microscopy (SEM) analysis of *C. albicans* morphology revealed that the *C. albicans* biofilm structure in the *S. mutans* MV-treated group was three-dimensional, with a large amount of biofilm extracellular matrix, and that *C. albicans* formed hyphal cells under biofilm-forming conditions (Figure 3).

However, there was no significant difference in the *C. albicans* 24-h growth kinetics under planktonic conditions between the *S. mutans* MV-treated group and the control group (Supplementary Figure 2A; *P* > 0.05). SEM analysis of *C. albicans* cells under planktonic conditions showed that the *C. albicans* cell morphologies in the *S. mutans* MV-treated and control groups were both yeast forms (Supplementary Figure 2B).

### *S. mutans* MVs Localize in to the *C. albicans* Biofilm Extracellular Matrix and Contribute to Biofilm Extracellular Matrix Production

**FIGURE 4:**
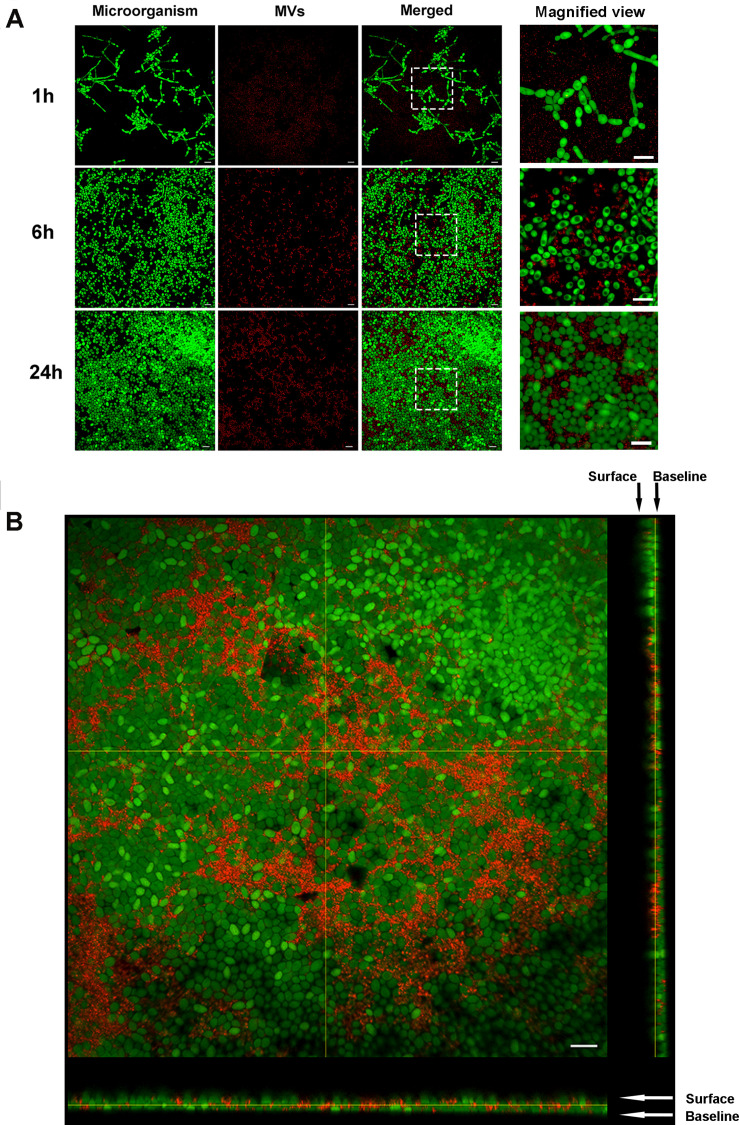
Fluorescent labeling of MVs and tracing. PKH26 (in red)-stained MVs were incubated with *C. albicans* at different time points. The *C. albicans* cells were stained with SYTO-9 (in green). **(A)** Representative CLSM images of *C. albicans* biofilms at 1, 6, and 24 h, the white boxes indicate the magnified viewing area. **(B)** Three-dimensional reconstructions of *C. albicans* 24-h-old biofilms. Magnification, 60×; scale bar, 20 μm. CLSM images showed that *S. mutans* MVs were located in the *C. albicans* biofilm extracellular matrix.

The isolated *S. mutans* MVs were stained with PKH26 (in red), and *C. albicans* cells were stained with SYTO-9. CLSM was used to observe the location of *S. mutans* MVs in *C. albicans* biofilm. After 1 h of *C. albicans* biofilm development, *C. albicans* started to transform from the yeast to hyphal form, and *S. mutans* MVs were located in intercellular spaces (Figure 4A). We monitored the fate of *S. mutans* MVs in *C. albicans* biofilms for 6 and 24 h, and no colocalization was observed between *S. mutans* MVs and *C. albicans* (Figure 4A). Furthermore, CLSM three-dimensional reconstruction images of *C. albicans* 24-h-old biofilms showed that *S. mutans* MVs were located in the *C. albicans* biofilm extracellular matrix (Figure 4B).

**FIGURE 5:**
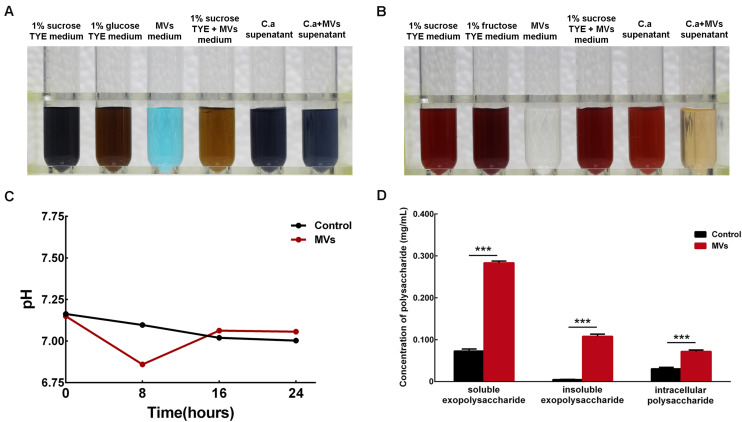
Monosaccharide, pH value and polysaccharide detection in *C. albicans* 24-h-old biofilms. **(A)** Benedict’s test of 1% sucrose TYE with *S. mutans* MVs containing reducing sugars (*C. a* supernatant was the supernatant of *C. albicans* biofilm culture with 1% sucrose TYE medium, *C. a* + MVs supernatant was the supernatant of *C. albicans* biofilm culture with 1% sucrose TYE culture and *S. mutans* MVs medium). **(B)** Seliwanoff’s test of 1% sucrose TYE with *S. mutans* MVs containing fructose. **(C)** pH measurements of the culture medium supernatants. **(D)** Polysaccharide detected by an anthrone-sulfuric acid colorimetric assay (The experiments were performed in three distinct replicates, and the data are presented as the means ± SD, ***P < 0.001 vs control group, using PBS as control group).

Benedict’s test was used to detect reducing sugars, such as glucose and fructose, in culture medium supernatant. The 1% sucrose TYE + MVs medium was brown, which indicated positive results for the presence of reducing sugars, while the supernatants from the *C. albicans* and *C. albicans* + MVs groups were both negative for reducing sugars (Figure 5A). This result indicated that *S. mutans* MVs can break down sucrose into reducing sugars and that *C. albicans* can effectively use reducing sugars. We also performed Seliwanoff’s test for the detection of ketose-like fructose. The results showed that 1% sucrose TYE + MVs medium was bright red, indicating the presence of fructose, while no fructose was present in the supernatant of the *C. albicans* + MVs group (Figure 5B), which was consistent with the results of Benedict’s test. The 1% sucrose TYE medium and *C. albicans* supernatant were bright red due to Seliwanoff’s reagent containing HCl and the HCl-mediated hydrolysis of sucrose into glucose and fructose. Taken together, these results demonstrated that *S. mutans* MVs contribute to sucrose metabolism in *C. albicans*. Furthermore, the pH of the culture medium supernatants was measured in a time-dependent manner (Figure 5C). The supernatant of *S. mutans* MV-treated group had a significant reduction by 0.24 in pH value compared to the control group at 8-h-old biofilm (Figure 5C; *P* < 0.001), while there was no significant difference between the *S. mutans* MV-treated group and the control group at 16-h-old and 24-h-old biofilm (Figure 5C; *P* > 0.05). We performed an anthrone-sulfuric acid colorimetric assay for the detection of soluble exopolysaccharide, insoluble exopolysaccharide and intracellular polysaccharide. All of these polysaccharides were significantly enriched in the *S. mutans* MV-treated group, and among them, the increase in insoluble exopolysaccharide by 22.85-fold was the most remarkable (Figure 5D; *P* < 0.001). Therefore, *S. mutans* MVs apparently contribute to the production of the *C. albicans* biofilm extracellular matrix.

### Gtfs of *S. mutans* MVs Promote *C. albicans* Biofilm Formation

**FIGURE 6:**
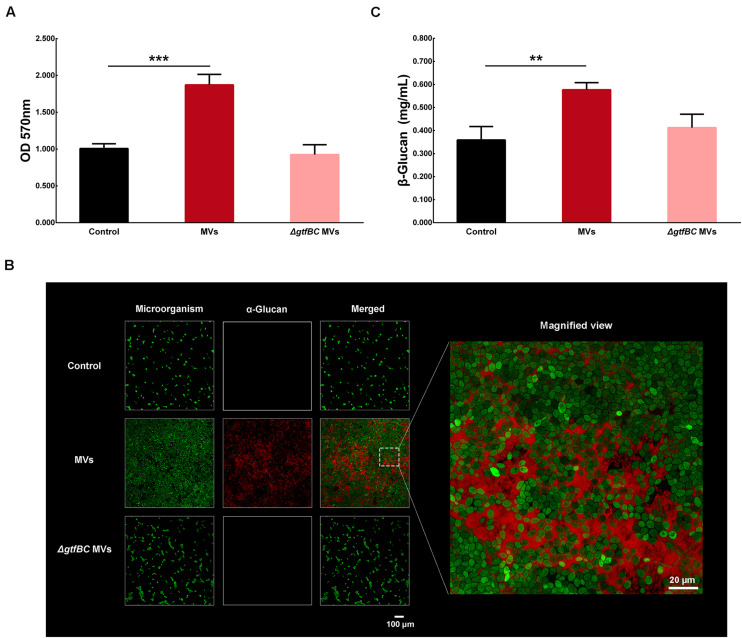
*C. albicans* biofilm formation by *S. mutans* MVs. **(A)** Crystal violet assay of *C. albicans* 24-h biofilm formation in conditions with *S. mutans* MVs and *S. mutans*Δ*gtfB*C mutant MVs. **(B)** CLSM images taken at 20 × magnification. α-Glucans were detected with an Alexa Fluor 647 dextran conjugate (in red), and the *C. albicans* cells were stained with SYTO-9 (in green). The white box indicated the magnified view area. **(C)** β-glucans concentration of *C. albicans* biofilm. The experiments were performed in three distinct replicates, and the data are presented as the means ± SD, ***P < 0.001, **P < 0.01 vs control group.

Gtfs are the primary proteins in *S. mutans* MVs (Senpuku et al., 2019), with GtfB and GtfC enzymes being involved in the production of α-glucans (Loesche, 1986). MVs from the *S. mutans* Δ*gtfBC* mutant were isolated by ultracentrifugation and added to culture medium to assess their effect on *C. albicans* biofilm development. Interestingly, the crystal violet assay results showed that *S. mutans* MVs significantly increased *C. albicans* biofilm formation compared to that observed in the control group (Figure 6A; *P* < 0.001), while *C. albicans* treated with *S. mutans* Δ*gtfBC* mutant MVs were not significantly different from those of the control group (Figure 6A; *P* > 0.05). These results indicated that *S. mutans* Δ*gtfBC* mutant MVs had no effect on *C. albicans* biofilm development and that Gtfs associated with *S. mutans* MVs promote *C. albicans* biofilm formation. We used an Alexa Fluor 647 dextran conjugate to incorporate into α-glucan during the *C. albicans* biofilm formation (Xu et al., 2014) and observed that α-glucan (in red) was detectable in *C. albicans* biofilms in the presence of *S. mutans* MVs, while no α-glucan synthesis was detected in the *S. mutans* Δ*gtfBC* mutant MV-treated and control groups (Figure 6B). Meanwhile, we measured the β-glucans secreted from *C. albicans*. β-glucans concentration of *S. mutans* MVs-treated group was significantly increased by 1.61-fold compared to that in the control group (Figure 6C; *P* < 0.01), while β-glucans concentration of *S. mutans* Δ*gtfBC* mutant MVs group was not significantly different from those of the control group (Figure 6C; *P* > 0.05). Taken together, these results suggested that Gtfs of *S. mutans* MVs involved in exopolysaccharide production significantly contribute to *C. albicans* biofilm formation.

### *S. mutans* MVs Increase the Expression of *Ndt80*, *Als1*, *Mnn9*, *Van1*, *Gca1*, *Big1*, and *Pmr1* in *C. albicans* Biofilms

Based on a number of genetic screens, *Ndt80*, *Brg1*, *Tec1*, *Rfx2*, *Efg1*, *Rob1*, *Gal4*, *Bcr1*, and *Flo8* were identified as a “core” set of regulators of *C. albicans* biofilm development (Nobile et al., 2012; Lohse et al., 2018). In addition, adherence and extracellular matrix production also play major roles in *C. albicans* biofilm development (Lohse et al., 2018). We examined the expression level of genes involved in *C. albicans* biofilm development regulation (*Ndt80*, *Brg1*, *Tec1*, *Rfx2*, *Efg1*, *Rob1*, *Gal4*, *Bcr1*, and *Flo8*), adhesion regulation (*Als1*, *Als2*, *Csh1*, *Eap1*, *Als3*, and *Hwp1*) and the production of extracellular matrix (*Mnn9*, *Van1*, *Gca1*, *Big1*, *Mnn1*, *Pmr1*, *Gca2* and *Gsc1*) (Lohse et al., 2018). RT-PCR results indicated that the expression levels of *C. albicans Ndt80*, *Als1*, *Mnn9*, *Van1*, *Pmr1*, *Gca1*, and *Big1* were significantly increased, while those of *Als3* and *Hwp1* were significantly decreased in the presence of *S. mutans* MVs compared to those observed under control conditions (Supplementary Figure 3; *P* < 0.05). Subsequently, the levels of these upregulated genes were assessed in the *S. mutans* MV-treated and *S. mutans* Δ*gtfBC* mutant MV-treated groups.

**FIGURE 7:**
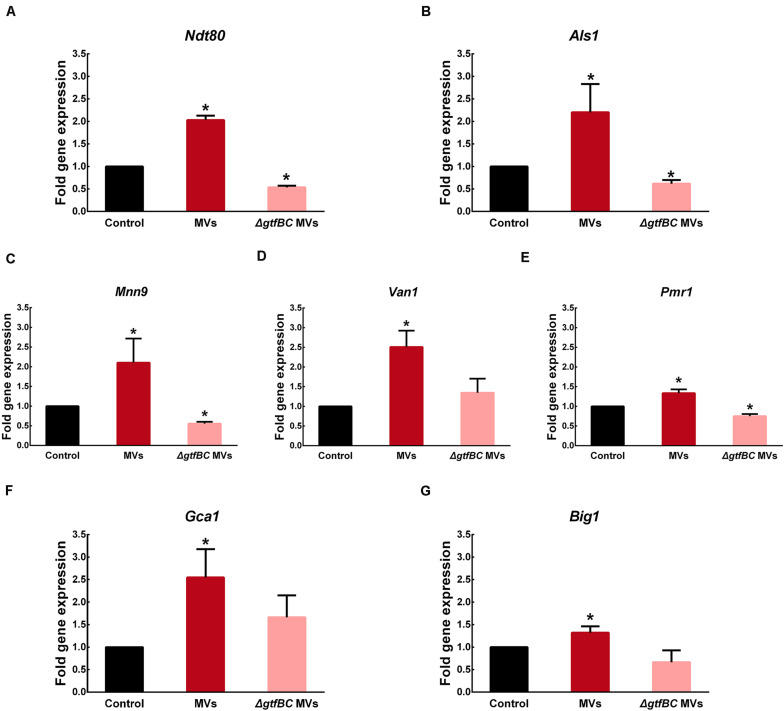
*S. mutans* MVs increase the relative gene expression of *C. albicans* biofilm-related transcriptional regulators **(A)** *Ndt80*, **(B)** *Als1*, **(C)** *Mnn9*, **(D)** *Van1*, **(E)** *Pmr1*, **(F)** *Gca1*, **(G)** *Big1*. The experiments were performed in three distinct replicates, and data are presented as the means ± SD, *P < 0.05 vs control group, using PBS as control group.

As one of core regulators of *C. albicans* biofilm development, *Ndt80* expression was significantly increased by 2.02-fold in the presence of *S. mutans* MVs compared to that observed under control conditions, while it was significantly decreased by 0.54-fold in the *S. mutans* Δ*gtfBC* mutant MV-treated group (Figure 7A; *P* < 0.05). Adhesion regulator *Als1* expression was significantly increased by 2.19-fold in the *S. mutans* MV-treated group and significantly decreased by 0.62-fold in the *S. mutans*Δ*gtfBC* mutant MV-treated group (Figure 7B; *P* < 0.05). These results indicated that *Ndt80* and *Als1* may play important role in Gtfs of *S. mutans* MVs in enhancing *C. albicans* biofilm development.

*Mnn9*, *Van1*, and *Pmr1* contribute to mannan production in *C. albicans* (Mitchell et al., 2015). In the *S. mutans* MV-treated group, the expression of *Mnn9*, *Van1*, and *Pmr1* was significantly increased by 2. 10-, 2.51- and 1.33-fold, respectively (Figures 7C–E; *P* < 0.05). In contrast, in the *S. mutans*Δ*gtfBC* mutant MV-treated group, *Mnn9* and *Pmr1* expression was significantly decreased by 0.55- and 0.75-fold, respectively (Figures 7C,E; *P* < 0.05), while *Van1* expression was not significantly different from that observed in the control group (Figure 7D; *P* > 0.05). *Gca1* and *Big1* regulate β-glucan synthesis in *C. albicans* (Mitchell et al., 2015). *Gca1* and *Big1* expression levels were significantly increased by 2.55- and 1.32-fold, respectively, in the *S. mutans* MV-treated group (Figures 7F,G; *P* < 0.05), but no significant difference in the expression of these genes was observed *S. mutans*Δ*gtfBC* mutant MV-treated group compared to that observed in the control group (Figures 7F,G. *P* > 0.05).

## Discussion

Membrane vesicles secretion is a universal phenomenon and most of bacteria, mycobacteria and fungi can produced it (Brown et al., 2015). Studies have demonstrated that *Bacillus subtilis* and *C. albicans* produced MVs under the planktonic condition and biofilm (Brown et al., 2014; Zarnowski et al., 2018). *S. mutans* is the third Gram-positive bacterium shown to produce MVs (Liao et al., 2014). We isolated *S. mutans* MVs from the planktonic condition, and observed that the morphology of *S. mutans* MVs was “cup-shaped” and that *S. mutans* MVs were distributed in different size ranges, which was consistent with the results of previous studies on MVs isolated from *S. mutans* and other microorganisms (Brown et al., 2014; Liao et al., 2014). For instance, the size distribution of *B. subtilis* MVs is between 50 nm and 150–250 nm (Brown et al., 2014), while MVs produced by *C. albicans* range between 50–100 nm and 350–450 nm in diameter (Vargas et al., 2015). Current evidence supports a role for the holin-endolysin pathway in the mechanism of Gram-positive bacterial MV biogenesis (Toyofuku et al., 2017; Toyofuku et al., 2019). MVs can be released through cell wall holes produced by endolysin (Toyofuku et al., 2017), and MVs might be reassembled and naturally recovered (Brown et al., 2014). We also had tried to isolated *S. mutans* MVs from the biofilm culture, and found that *S. mutans* also produced MVs under biofilm culture (Supplementary Figure 4). But it hard to purified *S. mutans* MVs from the biofilm culture due to the subproducts of *S. mutans* biofilm like glycoprotein and lipid.

*Candida albicans* biofilms, an important virulence factor, are formed by a structured mixture of different phenotypic cells (yeast, pseudohyphal, and hyphal) and extracellular matrix (Lohse et al., 2018). The results of our study showed that *S. mutans* MVs enhance *C. albicans* biofilm development but do not impact *C. albicans* growth under planktonic conditions, similar to the findings of previous research on *S. aureus* MVs (Im et al., 2017) in which *S. aureus* MVs were shown to influence the biofilm formation of other bacteria (*A. baumannii*, *E. faecium*, and *K. pneumoniae*) but did not affect the growth of these bacteria (Im et al., 2017). *S. mutans* MVs contain biologically active substances and promote oral microorganism biofilm formation, including that of *S. mutans*, *S. sanguinis*, *S. mitis*, *S. gordonii*, and *S. oralis* (Senpuku et al., 2019). However, in contrast to the results of the present study, a previous study reported that *S. mutans* MVs had no effect on *C. albicans* biofilm development (Senpuku et al., 2019). This discrepancy may be due to differences in the sucrose concentrations in culture medium and different culturing methods used. The Senpuku group used tryptic soy broth without dextrose supplemented with 0.25% sucrose to generate biofilms, while our group used tryptone-yeast extract medium supplemented with 1% sucrose. This indicated that the effect of *S. mutans* MVs on *C. albicans* biofilm development may be culture medium dependent. Kinds of culture medium including Sabouraud’s glucose broth with 1% sucrose, TYE with 1% sucrose, RPMI1640, artificial saliva solution supplemented with 1% sucrose and 10% fetal bovine serum employed to develop *C. albicans* biofilm (Xiao et al., 2018a; Yang et al., 2018; Li et al., 2019). TYE medium culture is used for the routine growth of *C. albicans* and similar to the oral environment, which is often used to investigate the cross-kingdom interaction between *S. mutans* and *C. albicans* (Ellepola et al., 2017; He et al., 2017; Kim et al., 2017). Sucrose is recognized as strong cariogenicity food in the world (Durso et al., 2014). Therefore, TYE with 1% sucrose culture medium is better simulate the oral caries environment. In addition, the inoculum was grown at 37°C under aerobic conditions with a shaking speed of 75 rpm for 90 min to promote cells adherence to solid surface. In the *S. mutans* MV-treated group, hyphal cells were observed under *C. albicans* biofilm-forming conditions, but no phenotypic transition was observed under planktonic conditions. This result indicated that *S. mutans* MVs induced *C. albicans* transformation from yeast to the hyphal form under biofilm-forming conditions. Yeast-filamentous growth is a typical phenotypic switching systems of *C. albicans* (Whiteway and Bachewich, 2007). Many types of environmental factors, such as pH, CO_2_, serum, and GlcNAc influence the *C. albicans* phenotypic transition (Cassone et al., 1985; Mock et al., 1990). The hyphae of hyphal cells can provide adhesion sites for other cells and increase biofilm thickness (Lohse et al., 2018), enhancing the virulence of *C. albicans*.

The biofilm extracellular matrix, as a physical scaffold, is a significant part of biofilms that can promote microorganism aggregation and adhesion (Lohse et al., 2018). The *C. albicans* biofilm extracellular matrix consists of glycoproteins, exopolysaccharides, lipoteichoic acid, and nucleic acids (Zarnowski et al., 2014) and plays an important role in the pathogenesis of ECC (Bowen and Koo, 2011). α-mannan and β-1,6-glucan are the predominant polysaccharides in *C. albicans* biofilm extracellular matrix, they form a mannan-glucan complex in biofilm extracellular matrix (Lohse et al., 2018). By itself, *C. albicans* is unable to efficiently utilize sucrose to synthesize extracellular matrix (Kim et al., 2017), but a large amount of biofilm extracellular matrix was formed in the *S. mutans* MV-treated group. Benedict’s and Seliwanoff’s tests demonstrated that *S. mutans* MVs decomposed sucrose into glucose and fructose and that *C. albicans* treated with these MVs could effectively utilize these monosaccharides. The supernatant of *S. mutans* MV-treated group had a significant reduction in pH value compared to the control group at 8-h-old biofilm. The metabolism of monosaccharides produces acid and promotes *C. albicans* hyphal morphogenesis (Han et al., 2011), which may partly explain why *C. albicans* in the *S. mutans* MV-treated group formed hyphal cells under biofilm-forming conditions. CLSM images showed that *S. mutans* MVs were located in the extracellular matrix of *C. albicans* biofilms. Anthrone-sulfuric acid colorimetric assay results showed that soluble exopolysaccharide, insoluble exopolysaccharide and intracellular polysaccharide levels were significantly increased in the *S. mutans* MV-treated group, with the increase in insoluble exopolysaccharide content being the most remarkable. A previous study reported that *Bacteroides* outer MVs can promote polysaccharide metabolism in the human intestine (Elhenawy et al., 2014). Outer MVs can be a “public goods” that promote resource acquisition and benefit the microbial community (Caruana and Walper, 2020). Altogether, these findings provide evidence that *S. mutans* MVs contribute to the production of *C. albicans* biofilm exopolysaccharides.

*Streptococcus mutans* Gtfs include GtfA, GtfB, GtfC, and GtfD and are important virulence factors in this bacterium (Loesche, 1986). GtfB and GtfC play major roles in sucrose metabolism. GtfB involved in the production of water-insoluble exopolysaccharide that largely consist of α-1,3-glucans, GtfC produce both water-insoluble and water-soluble glucans (Loesche, 1986). Recent studies have shown that Gtfs are the main proteins in *S. mutans* MVs (Senpuku et al., 2019). The results of the present study showed that MVs from an *S. mutans* Δ*gtfBC* mutant did not promote *C. albicans* biofilm formation. Moreover, the CLSM images revealed that no α-glucan synthesis occurred in the *S. mutans* Δ*gtfBC* mutant MV-treated group. Previous studies have indicated that an *S. mutans* Δ*gtfBC* mutant could not form a mixed biofilm with *C. albicans* (Falsetta et al., 2014). GtfB strongly binds to the *C. albicans* surface mannan to promote the formation of a glucan-rich matrix *in situ*, then enhances *C. albicans* aggregation and promotes *C. albicans* biofilm development (Gregoire et al., 2011; Hwang et al., 2015; Ellepola et al., 2017; Hwang et al., 2017). α-Glucans are polysaccharides produced by bacteria that can provide sites for microorganisms to adhere and promote the formation of cariogenic biofilms (Loesche, 1986). *C. albicans* cannot synthesize α-glucans by itself (Lohse et al., 2018), which was consistent with the remarkable increase in insoluble exopolysaccharide levels in the *S. mutans* MV-treated group. Taken together, these results indicated that Gtfs in *S. mutans* MVs are involved in α-glucan production and significantly contribute to *C. albicans* biofilm formation. Meanwhile, *C. albicans* secreted β-glucans were significantly increased in the *S. mutans* MVs-treated group, while those of *S. mutans* Δ*gtfBC* mutant MVs group was not significantly different from those of the control group. This finding indicated that the α-glucan produced by Gtfs in *S. mutans* MVs may impact the β-glucans secretion of *C. albicans*.

RT-PCR results showed that *Ndt80*, *Als1* gene expression was significantly increased in the *S. mutans* MV-treated group compared to that observed in the control group. *Ndt80* is a key regulator of *C. albicans* biofilm thickness and hyphal development (Nobile et al., 2012), and regulates the expression of adhesion genes (*Als3* and *Hwp1*) (Sellam et al., 2010). SEM imaging of the *S. mutans* MV-treated group showed that the *C. albicans* biofilm was a three-dimensional structure and that *C. albicans* formed hyphal cells under biofilm-forming conditions, which was consistent with the upregulation of *Ndt80*. *Als1* is involved in the regulation of *C. albicans* adhesion (Mayer et al., 2013). The results of a number of studies have indicated that *Als1* may play an important role in promoting aggregation in cross-kingdom interactions (Diaz et al., 2012; Xu et al., 2014), as it can enhance the co-aggregation of *S. oralis* and *C. albicans* (Xu et al., 2017), and *S. mutans* was shown to stimulate *C. albicans Als1* gene expression to augment biofilm formation (Ellepola et al., 2017). In addition, the results of the present study showed that the gene expression levels of *Mnn9*, *Van1*, *Pmr1*, *Gca1*, and *Big1* were upregulated in the *S. mutans* MV-treated group. *Mnn9*, *Van1*, and *Pmr1* contribute to mannan production, while *Gca1* and *Big1* regulate β-glucan synthesis (Mitchell et al., 2015), and the expression of genes was correlated with the synthesis of exopolysaccharide. Previous studies reported that *S. mutans*-derived GtfB binds to *C. albicans* surface mannan (Hwang et al., 2015), transcriptomic analysis of *S. mutans*–*C. albicans* mixed biofilms reveals that *S. mutans* enhances *C. albicans* carbohydrate metabolism and alters mannan and glucan production of *C. albicans* (Ellepola et al., 2019), and the *C. albicans* biofilm polysaccharide production results showed that exopolysaccharide levels and *C. albicans* secreted β-glucans were significantly increased in the *S. mutans* MV-treated group. These results are consistent with the observed upregulation in the expression of the abovementioned *C. albicans* genes. However, the changes in the expression of transcriptional regulators is not completely consistent with biological behavior. The expression of protein and the histone modification also influence the biological behavior (Manzoni et al., 2018). Therefore, the concrete mechanism of *S. mutans* MV on *C. albicans* genes, protein expressions need further researches.

However, the level of *Ndt80*, *Als1*, *Mnn9*, and *Pmr1* gene expression in the *S. mutans* Δ*gtfBC* mutant MV-treated group were significantly decreased. CLSM imaging of *S. mutans* Δ*gtfBC* mutant MVs group was different with that of control group. These results indicated other components of the *S. mutans* MVs also play a role in *C. albicans* biofilm formation. Emerging studies have found that *S. mutans* MVs is an effective method of releasing eDNA (Liao et al., 2014; Rainey et al., 2019), and both homologous and heterologous eDNA has a crucial effect on *C. albicans* biofilm formation (Sapaar et al., 2014; Hirota et al., 2017). It also has been demonstrated that competence-stimulating peptide and subproducts of *S. mutans* suppress *C. albicans* hyphal cell and biofilm formation (Jarosz et al., 2009; Barbosa et al., 2016). Therefore, the specific components of *S. mutans* MVs on *C. albicans* development is unclear and requires further research.

The mechanism of the cross-kingdom interaction between *S. mutans* and *C. albicans* is complex and unclear. Although we successfully isolated *S. mutans* MVs under planktonic condition, the *S. mutans* MVs isolated from *S. mutans* to *C. albicans* co-culture medium is more worthwhile. We have recognized the role of *S. mutans* MVs on *C. albicans* growth and biofilm development, the effect of *S. mutans* MVs on *S. mutans*–*C. albicans* mixed biofilm *in vitro* and *in vivo* remains unclear. Due to the experimental limitations that *S. mutans* and *C. albicans* both can produce MVs, it hard to purified *S. mutans* MVs from *S. mutans*–*C. albicans* co-culture medium. Meanwhile, the effect of *C. albicans* MVs on *S. mutans* growth and biofilm formation is unknown. Further studies are needed to investigate the role of MVs on the cross-kingdom interaction between *S. mutans* and *C. albicans*.

In summary, results of the present study showed that *S. mutans* MVs augmented *C. albicans* biofilm development but had no significant effect on *C. albicans* growth under planktonic conditions. *S. mutans* MVs promote *C. albicans* biofilm formation by enhancing biofilm exopolysaccharides synthesis. Furthermore, we discovered that Gtfs in *S. mutans* MVs involved in α-glucan production contribute to *C. albicans* biofilm formation. Altogether, the results of the present study increase our understanding of the function of *S. mutans* MVs and provides new insights into the cross-kingdom interactions between *S. mutans* and *C. albicans*.

## Materials and Methods

### Bacterial Strains and Culture Conditions

*Streptococcus mutans* UA159 (ATCC 700610, provided by the Guangdong Microbial Culture Collection Center), a *S. mutans* UA159 Δ*gtfBC* mutant (Gong et al., 2018) and *C. albicans* SC5314 (ATCC MYA−2876) (Liu et al., 2019) were used in the present study. *S. mutans* UA159 was grown in brain heart infusion (BHI; Difco, Detroit, MI, United States) medium at 37°C under anaerobic conditions (80% N_2_, 10% H_2_, and 10% CO_2_). *C. albicans* SC5314 was grown in Sabouraud’s glucose broth (SDB, HKM, Guangzhou, China) at 37°C under aerobic conditions with a shaking speed of 200 rpm. To induce *C. albicans* biofilm development, cells were cultured in tryptone-yeast extract (TYE, OXOID, Hampshire, United Kingdom) medium supplemented with 1% sucrose.

### Preparation and Characterization of *S. mutans* MVs

*Streptococcus mutans* MVs were isolated as described by Liao et al. (2014), with some modifications. Briefly, *S. mutans* strains were grown in 500 mL of BHI medium at 37°C for 16 h. Following centrifugation for 15 min at 4°C at 6,000 × *g* to remove cells, the cell-free culture supernatants were spun for 15 min at 4°C at 10,000 × *g* to remove cell debris. The resulting supernatants were filtered through 0.22-μm filters (Millipore, MMAS, United States) and then concentrated using a 100 kDa Amicon ultrafiltration system (Millipore, MMAS, United States). Subsequently, the concentrates were centrifuged at 100,000 × *g* for 70 min at 4°C and the pellets were washed once with sterile PBS before being resuspended in sterile PBS.

The MV yield was calculated by measuring protein concentration using a BCA assay (CWBIO, Beijing, China). For morphological analysis, TEM (H7650, Hitachi, Japan) was used to observe and identify the presence of MVs (Vargas et al., 2015). A 10 μL suspension of MVs was adhered to formvar/carbon-coated nickel TEM grids, negatively stained for 1 min with 3% uranyl acetate, washed with ddH_2_O and observed by TEM at an acceleration voltage of 80 kV. The size distribution and diameter of MVs were measured by NTA (NanoSight NS300, Malvern, United Kingdom), as previously described (Arab et al., 2019).

### *C. albicans* Biofilm Formation and Quantification

*Candida albicans* biofilm development was assessed according to the method of previous studies (Tan et al., 2016), with some modifications. Briefly, *C. albicans* was incubated in SDB at 37°C under aerobic conditions with a shaking speed of 200 rpm overnight, after which the cell density was adjusted to approximately 1 × 10^6^ CFUs/mL. Then, the inoculum was added to culture plates and grown at 37°C under aerobic conditions with a shaking speed of 75 rpm for 90 min. Then, unattached cells were removed by washing twice with sterile PBS, after which fresh 1% sucrose TYE medium was added, and the biofilm was grown at 37°C under aerobic conditions for 24–48 h.

A crystal violet assay was used to quantify *C. albicans* biofilm biomass according to optimized protocols (O’Toole, 2011). Briefly, the supernatants and planktonic cells were removed by washing three times with sterile PBS, after which absolute methanol was added to fix the biofilms for 15 min. The fixed biofilms were then stained with 0.1% (w/v) crystal violet for 5 min. After being washed three times with sterile PBS, crystal violet was extracted with 33% (v/v) glacial acetic acid, and the plates were maintained at 37°C for 30 min. Subsequently, the absorbance of the 33% glacial acetic acid solution was measured at 570 nm with a spectrophotometer (Tecan, Reading, Switzerland). The experiment was performed in three biological replicates and three technical replicates.

XTT reduction assays were used to evaluate *C. albicans* biofilm viability (Liu et al., 2017). An XTT (Sigma-Aldrich, St. Louis, MO, United States) solution (0.5 mg/mL in PBS) and a menadione (Sigma-Aldrich, St. Louis, MO, United States) solution (1 mM in acetone) were prepared and filter sterilized (pore size of 0.22 μm). Before each assay, the XTT solution was mixed with the menadione solution at a volume ratio of 50:1. After incubation, the supernatants and planktonic cells were removed by washing three times with sterile PBS. Then, 100 μL of the XTT-menadione solution was added. After incubating in the dark for 3 h at 37°C, the supernatants were transferred to a new 96-well plate and detected at 492 nm. The experiment was performed in three biological replicates and three technical replicates.

### Growth Curves of *C. albicans*

Individual colonies of *C. albicans* were added to 100 μL of double-strength SDB. Then, equal volumes of solutions with different concentrations of MVs or PBS (control group) were added and incubated at 37°C under aerobic conditions. Subsequently, 100-μL aliquots of the mixtures were removed at 2 h intervals for 24 h and used to measure the optical density of the microbial cultures at 600 nm. The experiment was performed in three biological replicates and three technical replicates.

### Analysis of *C. albicans* Biofilms by CLSM and SEM

*Candida albicans* biofilms were developed on cover glass for 24 h according to the method mentioned in section “*C. albicans* Biofilm Formation and Quantification.” For CLSM analysis, the supernatants and planktonic cells were removed by washing three times with sterile PBS, and the biofilms were stained with 2.5 μM SYTO-9 (Invitrogen Corp., Carlsbad, CA, United States) for 15 min at room temperature in the dark. Images were obtained using a Zeiss LSM780-Carl confocal laser scanning microscope, and the excitation wavelength used for SYTO-9 was 485/498 nm. CLSM images were collected from three different fields of three biological samples. An image analysis system (COMSTAT) was used to calculate the biovolume of *C. albicans* cells.

The structure of the *C. albicans* biofilm was observed by SEM (Quanta 400F-FEI, Eindhoven, Netherlands). Briefly, the supernatants and planktonic cells were gently removed by washing three times with sterile PBS, after which 2.5% (w/v) glutaraldehyde was added to fix biofilms overnight at 4°C. The fixed biofilms were then washed three times with sterile PBS and progressively dehydrated by an ethanol gradient (30, 50, 70, 90, and 100% for 15 min each). Then, the biofilms were washed three times with tert-butanol, dried by lyophilization and sputter coated with gold. Finally, the biofilms were observed at 1,000 × and 2,500 × magnification by SEM. The experiment was performed in 3 biological replicates.

### *S. mutans* MVs Labeling and Tracking

*Streptococcus mutans* MVs were labeled with PKH26 (mini26, Sigma, United States) according to the manufacturer’s instructions. Briefly, PKH26 was added to the MV-PBS solution at room temperature for 4 min in the dark, after which1% BSA (Phygene, Fuzhou, China) was added to bind excess dye. The mixture was centrifuged at 120,000 × *g* for 2 h at 4°C and then washed three times with sterile PBS. Then, the pellets were resuspended in sterile PBS for further use. The labeled MVs were incubated with *C. albicans* to develop biofilms at 37°C under aerobic conditions for 1, 6, and 24 h. Then, SYTO-9 was used to tag *C. albicans* cells as previously described and viewed by CLSM. The excitation wavelength used to observe PKH26 was 551/567 nm. The experiment was performed in three biological replicates.

### Testing for Monosaccharides and pH of Culture Medium Supernatants

Monosaccharide testing experiments were divided into six groups: 1% sucrose TYE medium (used as the negative control group), 1% glucose/fructose TYE medium (used as the positive control group), MV medium (used as the blank control group), 1% sucrose TYE + MVs (*S. mutans* MVs were added into 1% sucrose TYE medium), *C. a* supernatant (the supernatant of *C. albicans* biofilm culture with 1% sucrose TYE medium) and *C. a* + MVs supernatant (the supernatant of *C. albicans* biofilm culture with 1% sucrose TYE culture and *S. mutans* MVs medium). These supernatants were centrifuged for 15 min at 4°C at 6,000 × *g* and then filtered through 0.22-μm filters (Millipore, MMAS, United States) to remove cells. Benedict’s reagent (Leagene Biotechnology, Beijing, China) and Seliwanoff’s reagent (Leagene Biotechnology, Beijing, China) were used to test the glucose and fructose contents in the medium, respectively, according to the protocols described by Ellepola et al. (2019). The experiment was performed in three biological replicates and three technical replicates.

The pH of the culture medium supernatants measured by a pH electrode (Mettler Toledo, Zurich, Switzerland). The culture medium supernatants of the *S. mutans* MV-treated group and the control group were collected at 8 h intervals for 24 h, then centrifuged for 15 min at 4°C at 6,000 × *g* and then filtered through 0.22-μm filters (Millipore, MMAS, United States) to remove cells. The experiment was performed in three biological replicates and three technical replicates.

### Analysis of *C. albicans* Biofilm Polysaccharide

The production of *C. albicans* biofilm polysaccharide was measured by an anthrone-sulfuric acid colorimetric assay (Mao et al., 2016). Briefly, soluble exopolysaccharides, insoluble exopolysaccharides and intracellular polysaccharides were extracted from *C. albicans* biofilms by the ethanol precipitation method. Each polysaccharide was dissolved in 1 M NaOH, and four volumes of anthrone-sulfuric acid were added and heated for 6 min at 95°C, with dextrose anhydrate used as a standard. The experiment was performed in four biological replicates and three technical replicates.

Alexa Fluor 647 dextran conjugate (Invitrogen Corp., Carlsbad, CA, United States) can be incorporated into GTFs-derived α-glucan during biofilm development (Xu et al., 2014), but cannot incorporate with *C. albicans*-derived β-glucans (Falsetta et al., 2014). We observed the distribution of GTFs-derived α-glucan in *C. albicans* biofilms according to previously optimized protocols (Hwang et al., 2017). Briefly, *C. albicans* biofilms were grown in confocal dishes with 2.5 μM Alexa Fluor 647 dextran conjugate in the culture medium. After incubating for 24 h, SYTO-9 was used to label *C. albicans* cells as previously described, and the biofilms were viewed by CLSM (Olympus, FV3000, Japan). The excitation wavelength used to visualize the Alexa Fluor 647 dextran conjugate was 647/668 nm. The experiment was performed in three biological replicates.

β-glucans is a component of *C. albicans* biofilm exopolysaccharides (Lohse et al., 2018). Exopolysaccharides were extracted from *C. albicans* biofilms by the ethanol precipitation method. *C. albicans* secreted β-glucans were measured using the commercially available β-glucans Assay Kit (Megazyme, K-YBGL, Ireland) according to the manufacturer’s directions. The experiment was performed in three biological replicates and three technical replicates.

### Analysis of *C. albicans* Gene Expression by Real-Time PCR

For *C. albicans* biofilm cell collection, the supernatants and planktonic cells were removed, and sterile PBS was added to harvest cells by centrifugation at 12,000 rpm for 5 min at 4°C. Total RNA was extracted from cell pellets using RNAiso Plus (Takara Bio Inc., Otsu, Japan) according to the manufacturer’s instructions, and the purity (A260/A280) and concentration of RNA were determined using a NanoDrop 2000 spectrophotometer (Thermo Fisher Scientific, Pittsburgh, PA, United States).

RNA was reverse transcribed with a PrimeScript^TM^ RT reagent kit with gDNA Eraser (Takara Bio Inc., Otsu, Japan) following the manufacturer’s protocol (Huang et al., 2017). The sequences of primers used for real-time PCR are presented in Supplementary Table 1. Real-time PCR was used to assess nine master transcriptional regulators of *C. albicans* biofilm development (Lohse et al., 2018), including transcription factors related to the production of extracellular matrix and cell adhesion, while the housekeeping gene *Pma1* used as an internal control (Chau et al., 2004). Amplification and quantification of target RNA were performed in a LightCycler 480 Real-Time System using SYBR^®^ Premix Ex Taq^TM^ II (2×) (Takara Bio Inc., Otsu, Japan). The gene expression fold changes were calculated using the 2^–Δ^ ^Δ^ ^Ct^ method. The experiment was performed in three biological replicates and three technical replicates.

### Statistical Analyses

Data are represented as the mean ± standard deviations (SD) from at least three biological replicates and three technical replicates. Statistical analysis was performed using SPSS 20.0. The level of significance was analyzed by unpaired *t*-test and one-way ANOVA combined with a Student-Newman-Keuls (SNK) *post hoc* test. *P* < 0.05 was considered significant.

## Data Availability Statement

The raw data supporting the conclusions of this article will be made available by the authors, without undue reservation.

## Author Contributions

RW, YZ, and HL designed the research, co-wrote, and revised the manuscript. RW executed the experiments and analyzed the data. YT and YC provided technical and theoretical support. All authors read and approved the final manuscript.

## Conflict of Interest

The authors declare that the research was conducted in the absence of any commercial or financial relationships that could be construed as a potential conflict of interest.

## Supplementary Material

FIGURE S1 *C. albicans* 24-h biofilm formation in the presence of different concentrations of *S. mutans* MVs. **(A)** Crystal violet assay. **(B)** XTT assay (The experiments were performed in three distinct replicates, and the data are presented as the means ± SD, ^∗∗∗^*P* < 0.001 vs control group, using PBS as control group).

FIGURE S2 The effect of *S. mutans* MVs on *C. albicans* growth under planktonic conditions. **(A)** *C. albicans* 24 h growth kinetics; **(B)** Morphological characteristics of *C. albicans* grown under planktonic conditions for 24 h. Magnification 2,000 × ; scale bar, 5 μm.

FIGURE S3 Expression of *C. albicans* biofilm-related transcriptional regulators. **(A)** The nine master regulators required for biofilm development. **(B)** Regulators related to adhesion. **(C)** Regulators related to extracellular matrix production. The experiments were performed in three distinct replicates, and data are presented as the means ± SD, ^∗^*P* < 0.05, ^∗∗^*P* < 0.01 vs control group, using PBS as control group.

FIGURE S4 Negative staining TEM of *S. mutans* MVs from biofilm culture. The white arrows indicate the *S. mutans* MVs, Scale bar, 100 nm.

TABLE S1 Sequences of primers used for RT-PCR.
