# Supplementary material for: Streptococcus mutans Membrane Vesicles Harboring Glucosyltransferases Augment Candida albicans Biofilm Development
Source: Front Microbiol. 2020 Sep 11;11:581184. doi: 10.3389/fmicb.2020.581184 (PMC7517897; doi:10.3389/fmicb.2020.581184)
Supplement: FIGURE S1 — C. albicans 24-h biofilm formation in the presence of different concentrations of S. mutans MVs. (A) Crystal violet assay. (B) XTT assay (The experiments were performed in three distinct replicates, and the data are presented as the means ± SD, ∗∗∗P < 0.001 vs control group, using PBS as control group). [file Data_Sheet_1.docx]

Supplementary Material

# Supplementary Figures and Tables

## Supplementary Figures


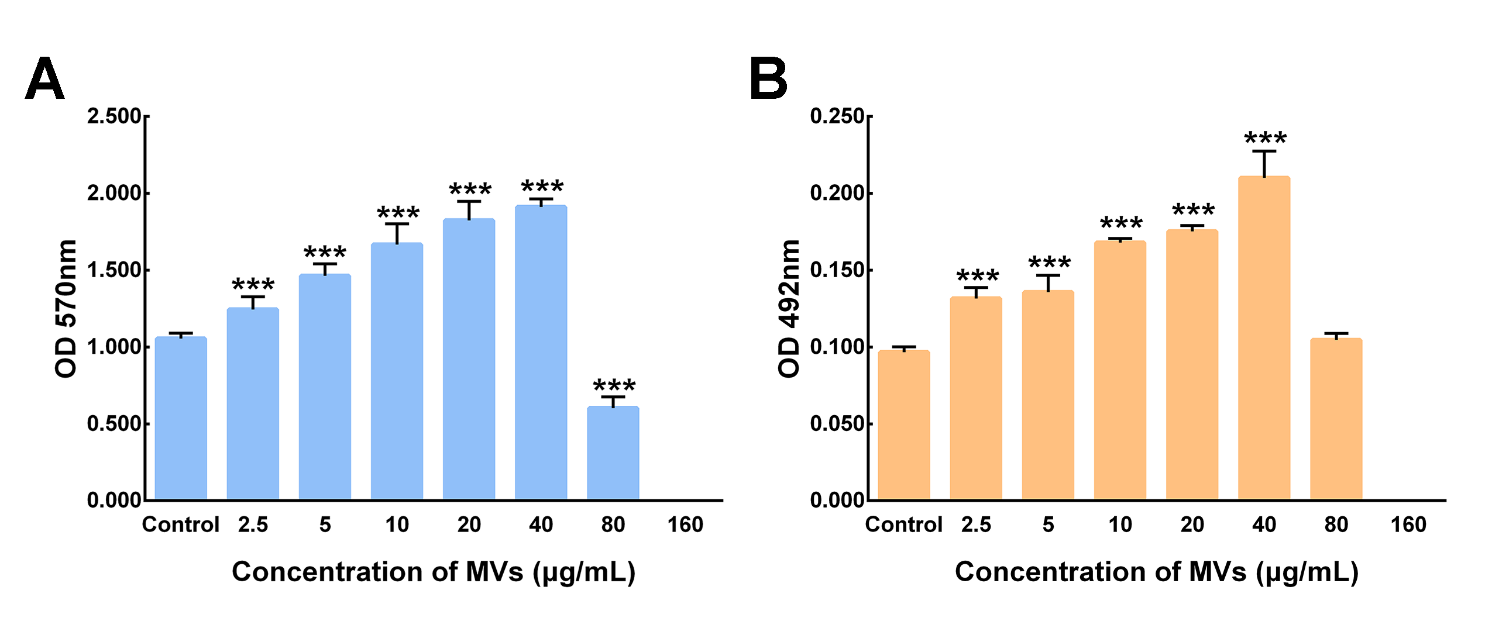


**Supplementary Figure 1.** *C. albicans* 24-h biofilm formation in the presence of different concentrations of *S. mutans* MVs. (A) Crystal violet assay. (B) XTT assay. (The experiments were performed in 3 distinct replicates, and the data are presented as the means ± SD, **^***^**: *P* < 0.001 vs. control group, using PBS as control group.


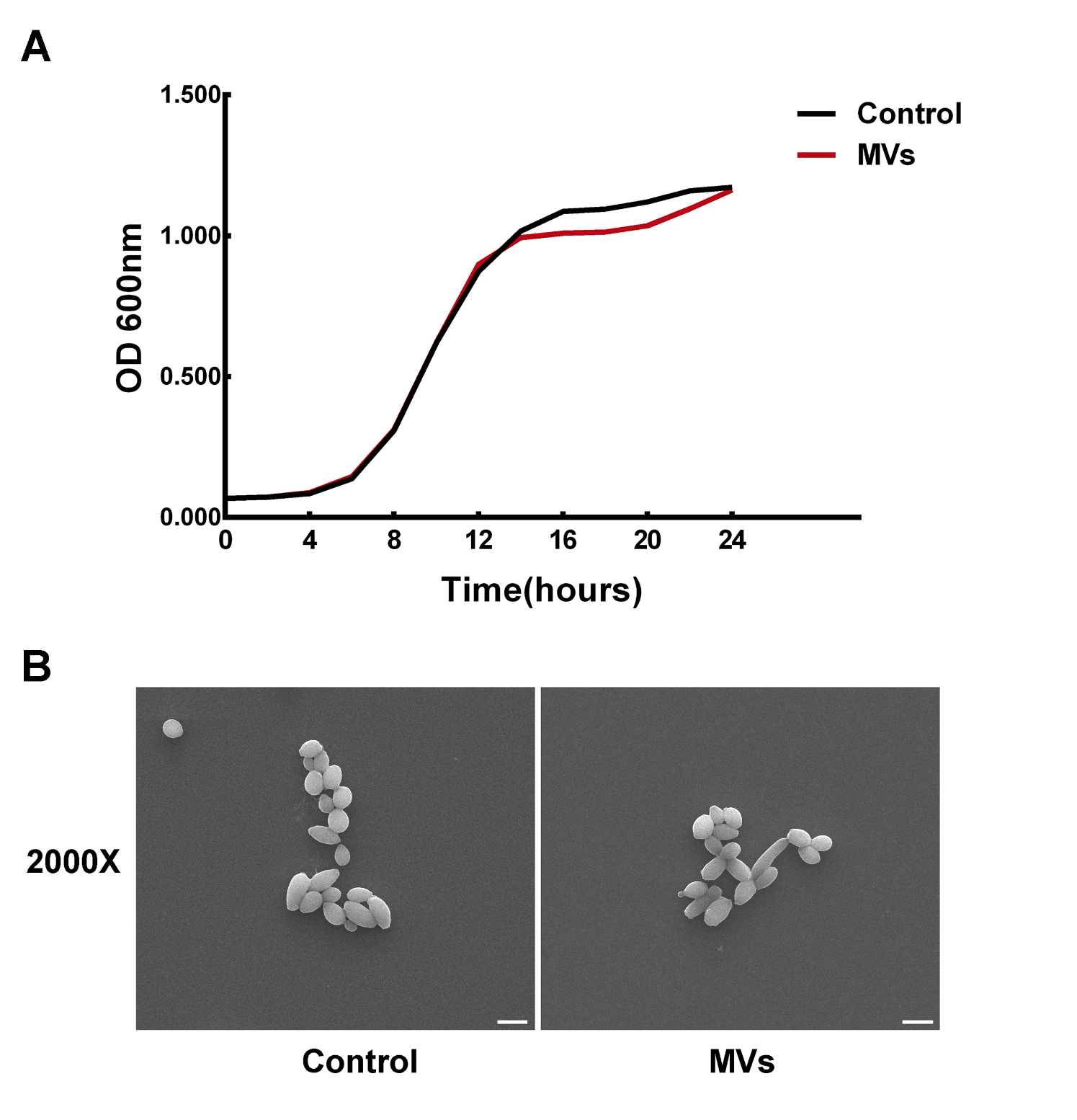


**Supplementary Figure 2** The effect of *S. mutans* MVs on *C. albicans* growth under planktonic conditions. (A) *C. albicans* 24 h growth kinetics; (B) Morphological characteristics of *C. albicans* grown under planktonic conditions for 24 h. Magnification 2,000×; scale bar, 5 μm.


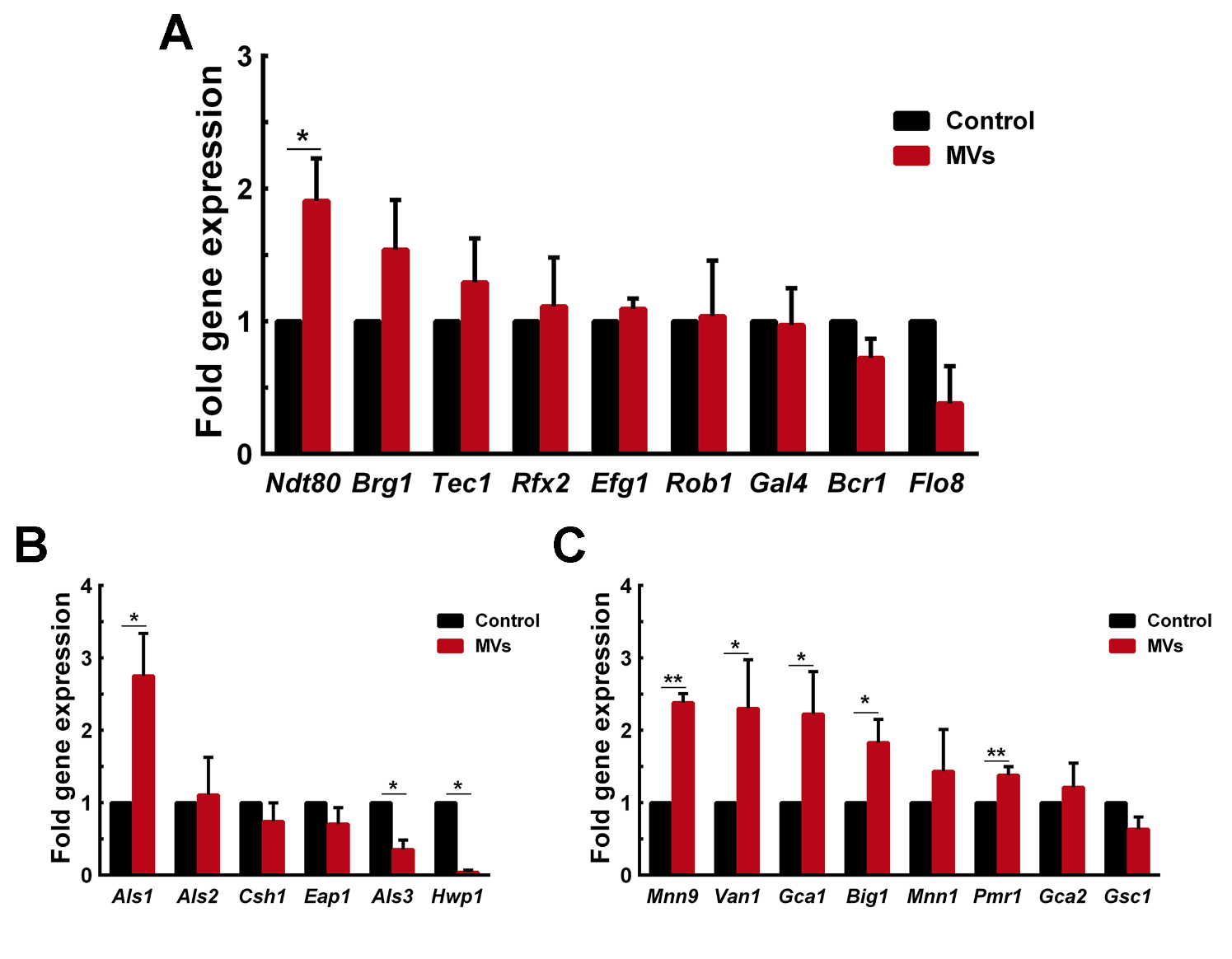


**Supplementary Figure 3** Expression of *C. albicans* biofilm-related transcriptional regulators. (A) The nine master regulators required for biofilm development. (B) Regulators related to adhesion. (C) Regulators related to extracellular matrix production. The experiments were performed in 3 distinct replicates, and data are presented as the means ± SD, **^*^**: *P* < 0.05, **^**^**: *P* < 0.01 vs. control group, using PBS as control group.


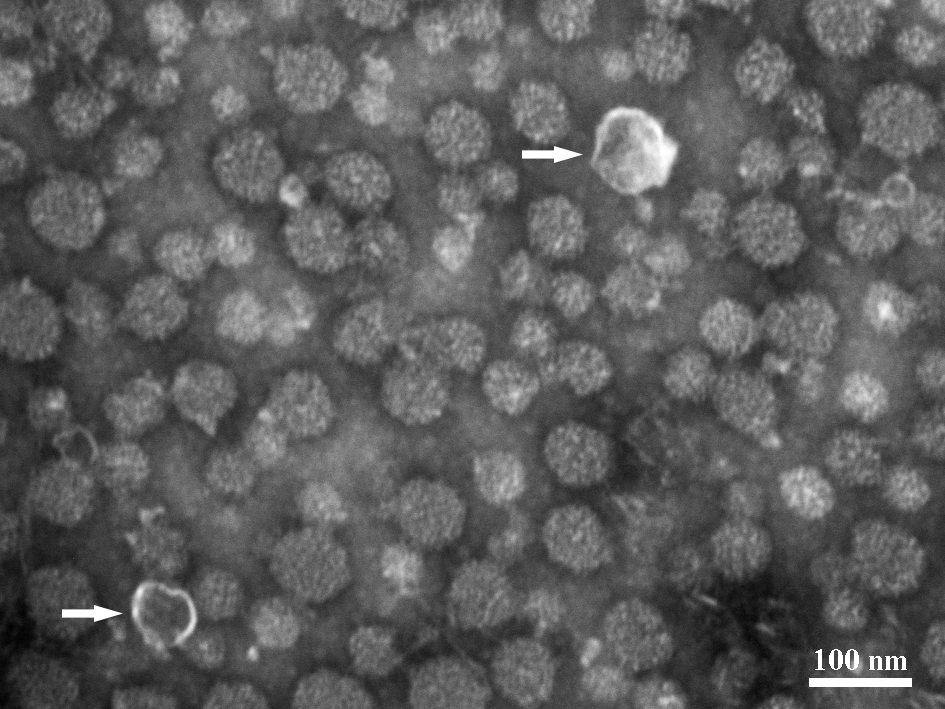


**Supplementary Figure 4** Negative staining TEM of *S. mutans* MVs from biofilm culture. The white arrows indicate the *S. mutans* MVs, Scale bar, 100 nm.

## Supplementary Tables

**Supplementary Table 1** Sequences of primers used for RT-PCR.

| **Target** | **Primer Sequence (5’-3’)** |
| --- | --- |
| ***Pma1*** | FW- TTGCTTATGATAATGCTCCATACGA  RV- TACCCCACAATCTTGGCAAGT |
| ***Ndt80*** | FW- AATCGAGGACGAGGACTTGC  RV- GTTGGGGTGTTTGTAGCCCT |
| ***Bcr1*** | FW- TGGGTGGTGGTCAAGAACAA  RV- TGCCAATGGTTCGGGTCTTC |
| ***Rfx2*** | FW- AACCCGTGTCGACTGGTAAC  RV- TTCGGAGTCAACACAGCCTC |
| ***Flo8*** | FW- CATTGGCACCAACTCAAGCC  RV- CCTGGGCCACTTACACTGAG |
| ***Rob1*** | RV- TTCTTGTGGTTGTGGTTCGTC  FW- AGCCAAAACATGAATACCACG |
| ***Gal4*** | FW- AGTGAAGTTGGAGCCGTCAG  RV- ACAGTTTCATCGACCACACCT |
| ***Tec1*** | RV- AGTAGGTGGAACAAAAGTGCC  FW- GCTCAGTAGCTTCACAACTGC |
| ***Efg1*** | FW- TATTACCAGGGTGGTGCTGC  RV- TGTTGGGGTGAAGGGTGAAC |
| ***Brg1*** | FW- GGGTTATTCCACGCTAAATTG  RV- TATTCTTCGACCGTTCCTCCC |
| ***Mnn9*** | FW- TGGGTCTTATGGTTGGATGCAG  RV- GGTGGACATTAGCGGAAACG |
| ***Mnn1*** | FW- CGGATGGTTATGGGAGTCGG  RV- CTCCGTGCCTACCCAAATGT |
| ***Pmr1*** | FW- AGCATGGTCTCCAACCACAG  RV- TGGGACAATCGATCGCAAGG |
| ***Van1*** | FW- AGGCAATCCACCAACACTGA  RV- TGTCGATATTGGCACGAGCA |
| ***Big1*** | RV- ATGGAAGAAACCCCAGCTTCG  FW- TGGATTCCAATTTGGTTCTACTTTG |
| ***Gsc1*** | RV- GAGTCGTCGTATGCTATTTTTGGTG  FW- TCCAACAATCGACACAGCAT |
| ***Gca1*** | FW- CCACCATACGCAATCAATAACA  RV- GCTTCGTAAATGGCACGTTCT |
| ***Gca2*** | FW- AACAAGCAGCAGTAGCGCC  RV- CATTGGCGTCAACAGCAGTATC |
| ***Als1*** | FW- AGAACTGATTTGCAGTGATGG  RV- TGAGGATTCATTGCTATCTGG |
| ***Als2*** | FW- GGGTTCACAATTGGCAGTGG  RV- CGATAACCAGCGGGGACATT |
| ***Csh1*** | FW- GGTTGGCACCAATTCATCTCC  RV- CGACACAAAACACCACCACC |
| ***Eap1*** | FW- TGTGATGGCGGTTCTTGTTC  RV- GGTAGTGACGGTGATGATAGTGA |
| ***Als3*** | FW- GCAACGTGCACCTTTCACAT  RV- TCGCGGTTAGGATCGAATGG |
| ***Hwp1*** | FW- TGAACCTTCCCCAGTTGCTC  RV- ATAGCACCACTTGAGCCAGC |
